# Primary Sternal Leiomyosarcoma

**DOI:** 10.1016/j.atssr.2024.07.006

**Published:** 2024-07-26

**Authors:** Marcus Rossi, Frank DiSilvio, Joseph Sahagun, David Ridder, Tyler Evans, Richard Anderson

**Affiliations:** 1 Department of Surgery, University of Illinois College of Medicine Peoria, Peoria, Illinois

## Abstract

We report the case of a primary leiomyosarcoma of the sternum in a 70-year-old man that was discovered incidentally during prostate cancer staging with positron emission tomography combined with computed tomography. Interventional radiology biopsied the lesion; pathologic examination showed spindle cells, indicating probable leiomyosarcoma. No primary site was found on contrast computed tomography, thus suggesting that the tumor was primary rather than metastatic. Given the tumor’s location and mortality risk, the patient underwent a sternotomy and reconstruction with methyl methacrylate, followed by an uneventful recovery. This case underlines a rare leiomyosarcoma presentation, constituting less than 0.7% of all primary malignant bone tumors, noted for its unusual location and rarity.

Primary leiomyosarcomas are relatively uncommon, with an incidence of <0.7% of all primary malignant bone tumors and soft tissue sarcomas.^1^ Excision of these tumors is highly recommended because of their associated poor prognosis, with a 35% overall survival rate.^2^ This report describes a case of primary leiomyosarcoma of the sternum, which was found incidentally in a patient undergoing a workup for prostate cancer staging and treatment planning.

We present the case of a 70-year old man with a past medical history of hypertension, hyperlipidemia, and chronic heart failure, as well as newly diagnosed prostate cancer, who had an incidental finding beneath the sternum while undergoing positron emission tomography (PET) combined with computed tomography (CT) for staging and treatment planning (Figure). Of note, the patient reported intermittent left-sided chest pain lateral to the sternum for almost a year but was never followed up. Interventional radiology performed a biopsy using a 17-gauge guider needle and obtained 3 separate 18-gauge core biopsy samples. The pathology report labeled the specimen as an atypical smooth muscle neoplasm, positive for smooth muscle actin (SMA) and desmin and negative for S100 and Mart 1, consistent with metastatic leiomyosarcoma. The report ruled it metastatic rather than primary because of the rarity of primary smooth muscle tumors. The patient had CT of the abdomen and pelvis with contrast enhancement to establish the origin of the leiomyosarcoma, but it was significant only for lytic sternal lesions and an enlarged prostate. The source of the leiomyosarcoma could not be ascertained. Throughout this course, the patient saw a cardiothoracic surgeon who recommended interventional radiology wire localization followed by sternal resection with pectoral advancement in coordination with plastic surgery.

**Figure:**
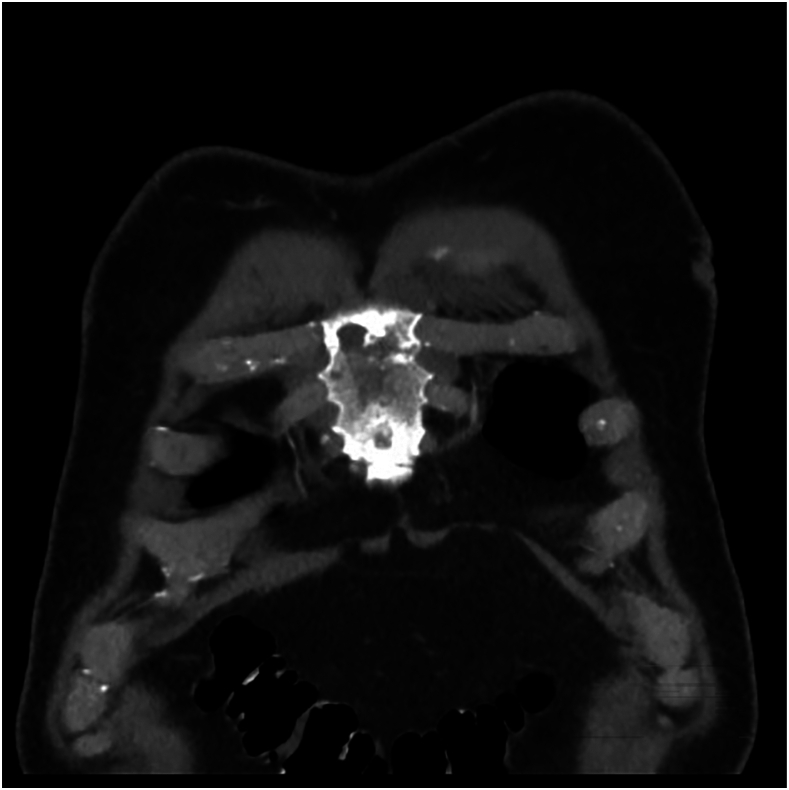
Positron emission tomography with computed tomography demonstrating lytic destruction of the sternum.

## Operative Procedure

### Sternal Resection

The procedure started with a midline sternotomy incision. After identifying and pulling a preoperative wire through the epidermis, the pectoralis major muscles were detached using electrocautery. A mass slightly to the right of the midline, appearing cartilaginous and bony, was exposed and biopsied, confirming a spindle cell neoplasm. Dissection continued until the tumor’s margins were clear. Windows were created in the intercostal spaces above and below the mass for surgical access. The sternum was segmented into upper and lower halves; the ribs on the right were divided with bone shears, during which the right internal mammary artery was transected and clipped. Attempts to preserve the left internal mammary artery were unsuccessful because of the location of the mass. The left ribs were also divided, and a 10 cm × 10 cm section of the sternum was removed. The specimen, initially showing negative fatty margins, was sent for pathologic examination.

### Sternal Reconstruction

A sterile polypropylene (Prolene, Ethicon) mesh with methyl methacrylate was placed in the defect and secured with interrupted 0-0 poly(ethylene, terephthalate) (Ethibond, Ethicon) sutures. The pectoralis major muscles were dissected to their origins and then repositioned to cover the mesh, thereby ensuring that thick skin and fat remained for perfusion. The muscles were attached to the mesh and surrounding ribs to achieve complete defect coverage. Two 15F Blake (Ethicon) drains were placed and the site was closed in layers, topped with an incisional strip wound vacuum-assisted closure and bilateral 16F chest tubes. The patient’s recovery was uneventful, and he was discharged on postoperative day 4 with plans for oncologic follow-up because of positive margins in the final pathology report.

## Comment

The case highlights an unusual presentation of leiomyosarcoma that was found incidentally on imaging. From our literature review, there are cases of primary and secondary chest wall tumors, but leiomyosarcomas typically develop in the uterus, gastrointestinal tract, and soft tissues. Therefore, clinical examination is essential to distinguish a primary tumor from a metastatic tumor.^3^^,^^4^ According to the abdominal and pelvis CT and PET-CT, there was no evidence of another site in our patient. It is challenging to distinguish malignancy from primary chest wall tumors solely on the basis of roentgenograms.^5^ Therefore, a biopsy of the lesion must be performed to rule out leiomyosarcoma, given its poor prognosis. On pathologic examination, spindle cell malignancy that stains positively for vimentin and SMA makes leiomyosarcoma more likely. In our case, the biopsy was positive for SMA and desmin, negative for other markers leading to the conclusion that the tumor was a leiomyosarcoma.

The treatment of choice for leiomyosarcoma of the bone is surgical resection with wide margins. The treatment within the literature varies by chemotherapy first and then resection, resection only, or resection followed up with chemotherapy, but the most successful was sternotomy with reconstruction.^6^ It is imperative to remove the tumor because approximately 50% of patients succumb to metastasis within 33 months, with death in most cases related to lung metastasis.^4^ In our patient, the mass was located in the sternum with the possibility of involving the inferior vena cava, causing compromise. Chen and colleagues^7^ reported that smooth muscle tumors involving the inferior vena cava require more aggressive surgical interventions and chemoradiation, but the overall prognosis is dismal. Intraoperatively, the lesions extended laterally to the patient’s left side, involving part of the first 2 ribs, which were not seen on the original PET-CT. Given the presentation, this case required a more extensive resection and sacrifice of the left internal mammary artery because of tumor extension. A 5- to 10-cm margin should be ensured to decrease the rate of tumor recurrence.^8^ After resection, the defect was 10 cm x 10 cm, and reconstruction consisted of methyl methacrylate with muscle flaps. Even after surgery, our patient had positive margins on pathologic examination, requiring follow-up with oncology for possible chemotherapy and radiation.

In conclusion, primary presentation of leiomyosarcoma is rare, and a diagnosis of leiomyosarcoma should always be considered when dealing with any chest wall malignancy. If possible, a biopsy should be obtained to rule out the tumor because of its overall high rate of metastasis and poor prognosis. Depending on the location, these tumors may be silent. In this case, the patient had a sternal leiomyosarcoma causing intermittent chest pain for about a year. The primary concern is that if the tumor continued to grow, it would eventually compress the mediastinal structures and put the patient at risk for obstructive shock. The first-line treatment involves resection of the mass, with chemoradiation as an adjunct to surgery if specimen margins are positive on pathologic examination.
